# Accurate target identification for *Mycobacterium tuberculosis* endoribonuclease toxins requires expression in their native host

**DOI:** 10.1038/s41598-019-41548-9

**Published:** 2019-04-11

**Authors:** Melvilí Cintrón, Ju-Mei Zeng, Valdir C. Barth, Jonathan W. Cruz, Robert N. Husson, Nancy A. Woychik

**Affiliations:** 1 Department of Biochemistry and Molecular Biology, Rutgers University, Robert Wood Johnson Medical School, Piscataway, NJ 08854 USA; 2 Member, Rutgers Cancer Institute of New Jersey, Piscataway, 08854 USA; 3 Division of Infectious Diseases, Boston Children’s Hospital/Harvard Medical School, Boston, MA 02115 USA

## Abstract

The *Mycobacterium tuberculosis* genome harbors an unusually high number of toxin-antitoxin (TA) systems. These TA systems have been implicated in establishing the nonreplicating persistent state of this pathogen during latent tuberculosis infection. More than half of the *M. tuberculosis* TA systems belong to the VapBC (virulence associated protein) family. In this work, we first identified the RNA targets for the *M. tuberculosis* VapC-mt11 (VapC11, Rv1561) toxin *in vitro* to learn more about the general function of this family of toxins. Recombinant VapC-mt11 cleaved 15 of the 45 *M. tuberculosis* tRNAs at a single site within their anticodon stem loop (ASL) to generate tRNA halves. Cleavage was dependent on the presence of a GG consensus sequence immediately before the cut site and a structurally intact ASL. However, in striking contrast to the broad enzyme activity exhibited *in vitro*, we used a specialized RNA-seq method to demonstrate that tRNA cleavage was highly specific *in vivo*. Expression of VapC-mt11 in *M. tuberculosis* resulted in cleavage of only two tRNA isoacceptors containing the GG consensus sequence, tRNA^Gln32-CUG^ and tRNA^Leu3-CAG^. Therefore, our results indicate that although *in vitro* studies are useful for identification of the class of RNA cleaved and consensus sequences required for accurate substrate recognition by endoribonuclease toxins, definitive RNA target identification requires toxin expression in their native host. The restricted *in vivo* specificity of VapC-mt11 suggests that it may be enlisted to surgically manipulate pathogen physiology in response to stress.

## Introduction

The bacterial pathogen that causes tuberculosis, *M. tuberculosis*, is able to evade the immune system and persist within its host for extended periods of time as a latent infection. Elucidation of the molecular mechanisms that underlie the latent state of *M. tuberculosis* is essential for developing more effective therapeutics for latent tuberculosis infection.

*The M. tuberculosis* genome harbors ~90 TA systems of which 50 belong to the VapBC family^1–5^. All VapC toxins contain a conserved catalytic PilT N-terminus (PIN) domain^1,2^; an intact PIN domain is essential for VapC toxicity and nuclease activity^6^. However, the stresses that trigger VapC toxin activities during infection are not known. In fact, the precise physiological roles of these 50 VapC toxins in *M. tuberculosis* infection and virulence are not well understood, although proposed to reduce protein synthesis^7^. It is also a conundrum why this pathogen harbors so many VapBC TA systems if collectively their only role is to simply reduce translation. To begin to tackle these broad and complex questions, we and others have been systematically studying their enzymatic activity as a first step toward understanding how they may influence the course of tuberculosis infections and reduce the efficacy of antituberculars.

RNA cleavage by members of the *M. tuberculosis* VapC-family of toxins has been reported using one of two general approaches. The first *in vitro* approach involves incubation of recombinant toxin with synthetic tRNAs^8,9^, synthetic RNAs^10–13^, or total RNA isolated from *M. tuberculosis*^8^. The second approach involves ectopic expression of the toxin in alternate rapidly growing hosts that do not require biosafety containment (*Escherichia coli* and *M. smegmatis*)^4,7,8,11–14^. Both of these approaches appeared to be reliable because only one or a few RNA targets were identified within the total RNA pool. Therefore, they are routinely employed to identify and characterize *M. tuberculosis* VapC toxin targets.

The enzymatic activities of 13 of the 50 *M. tuberculosis* VapC toxins have been studied to varying degrees^6–8,14^. Two of these 13 VapC toxins specifically target 23S rRNA at the sarcin-ricin loop^7,14^. The proposed RNA targets for the other 11 *M. tuberculosis* VapC toxins studied previously by Winther *et al*.^7^—including VapC-mt11 (aka VapC11, Rv1561) that is the focus of our study presented here—are derived from an RNA-VapC interaction screen using an *M. smegmatis* host.

We performed a thorough characterization of the enzymatic properties of the *M. tuberculosis* VapC-mt11 TA toxin *in vitro* and *in vivo* as well as its effect on protein synthesis and mycobacterial growth. We demonstrate that the *M. tuberculosis* VapC-mt11 toxin exhibits highly precise target specificity only when expressed in its natural *M. tuberculosis* host, in striking contrast to its much broader spectrum tRNase activity *in vitro*. These results also differ from those of Winther *et al*.^7^ and serve as a useful guide for the benefits and limitations of the array of approaches implemented by those studying endoribonuclease TA toxins.

## Results

### VapC-mt11 expression causes growth arrest in both *M. smegmatis* and *M. tuberculosis*

**Figure 1:**
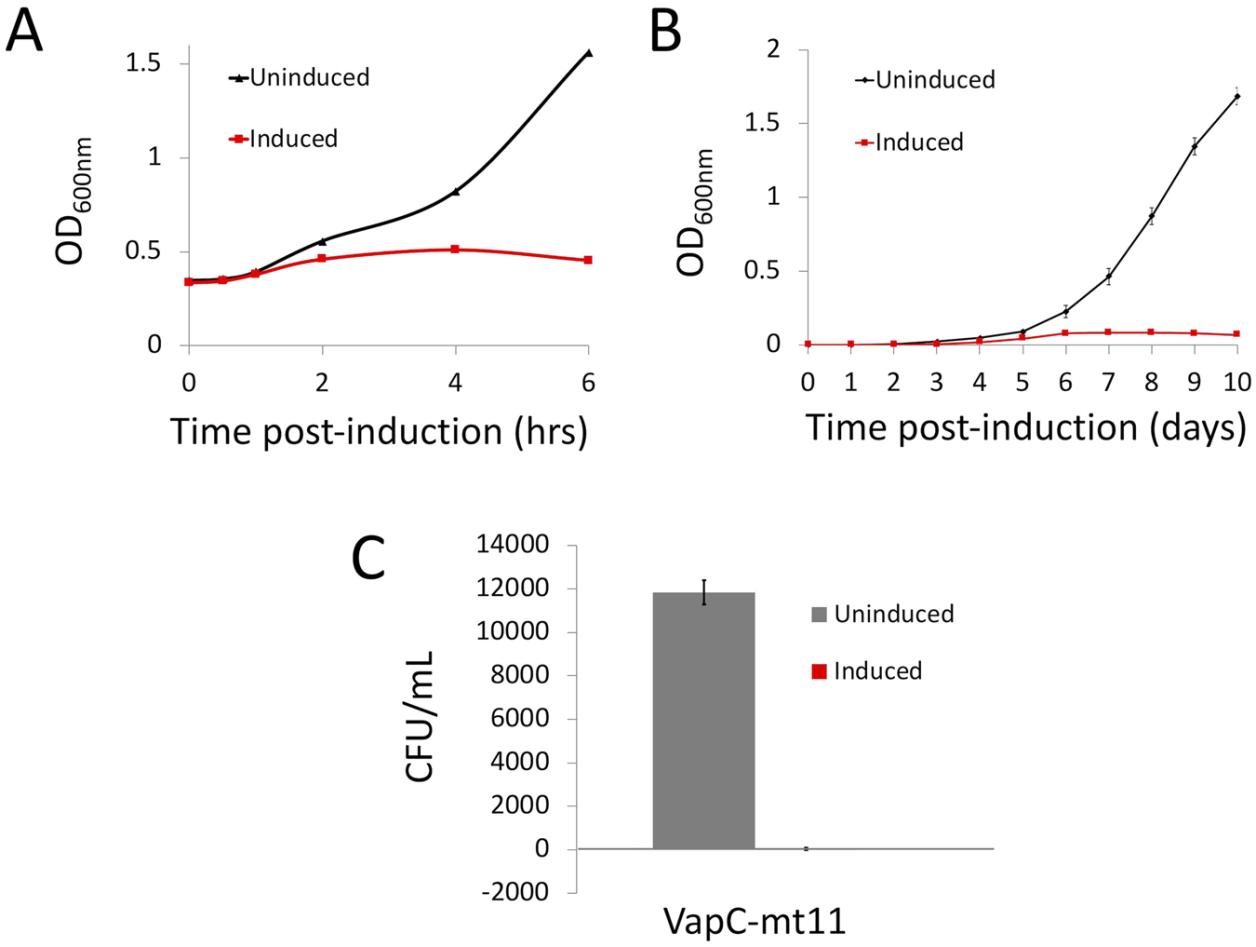
Growth inhibition by VapC-mt11. (**A**) *M. smegmatis* mc^2^155 harboring the anhydrotetracycline (ATc) inducible vector pMC1s with VapC-mt11 was grown at 37 °C in 7H9 + TW80 + AND supplemented with 25 ug/mL kanamycin. Cultures were split and one culture was induced by the addition of 200 ng/mL ATc. Time points for uninduced (♦) and induced () samples were collected up to 6 h. Growth curve shown is representative of the trend documented in three independent experiments. (**B**) *M. tuberculosis* H37Rv transformed with pMC1s-VapCmt11 were grown at 37 °C in 7H9-TW80-ADN medium containing 30% spent medium and supplemented with 25 ug/mL kanamycin. Cultures were separated into uninduced (▲) and induced (). Induced cultures were supplemented with 200 ng/ml ATc and additional ATc was added every 48 h to maintain the ATc concentration between 100 and 250 ng/ml. Data points represent the average of three independent experiments; error bars represent the S.D. (**C**) Plate efficiency assay for *M. tuberculosis* H37Rv cells harboring pMC1s-VapC-mt11 between uninduced (gray) and induced (red). Transformants were plated on 7H9 + TW80 + AND agar with 25 ug/mL kanamycin with or without 500 ng/mL of ATc and incubated for 3 weeks at 37 °C. Error bars represent the S.D. for biological and technical replicates.

*M. tuberculosis* H37Rv and *M. smegmatis* mc^2^155 cells were transformed with a VapC-mt11-containing anhydrotetracycline (ATc) inducible vector (pMC1s^15^). For *M. smegmatis*, VapC-mt11 expression resulted in growth arrest 2 h post-induction, which was sustained through the length of the growth profile (Fig. 1A). Likewise, VapC-mt11 expression arrested growth in *M. tuberculosis*. In contrast to the uninduced control, the OD_600_ for cells expressing VapC-mt11 remained near zero for the duration (10 days) of the growth profile (Fig. 1B). In concordance with the growth profile, the average CFU/ml recovered when *M. tuberculosis* cells were plated in the presence of ATc inducer was 50 CFU/ml compared to 12,000 CFU/ml from the uninduced control (Fig. 1C). These results were consistent with the strong growth inhibition we observed when VapC-mt11 was expressed in *E. coli* cells^6^.

### VapC-mt11 inhibits translation

**Figure 2:**
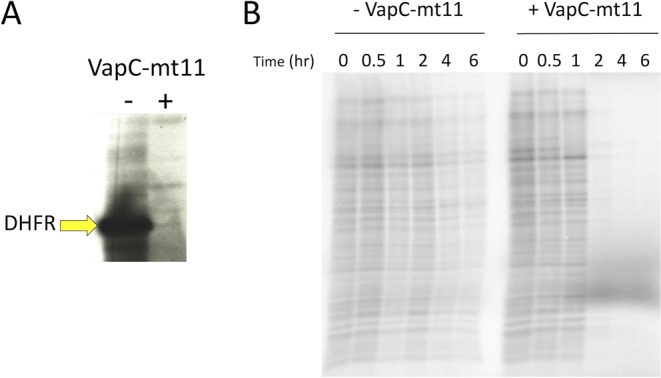
VapC-mt11 inhibits translation *in vitro* and in *M. smegmatis*. (**A**) The PURExpress translation reaction was incubated with (+) or without (−) VapC-mt11. Production of the control DHFR template was assayed (yellow arrow). Uncropped image shown in Supplementary Information Fig. 1 (**B**) [^35^S]-Methionine incorporation in *M. smegmatis* mc^2^155 cells grown to exponential phase and split into uninduced (−VapC-mt11) and induced (+VapC-mt11) cultures. Cell aliquots were collected for up to 6 h post induction. Equivalent amounts of cell lysate (resuspended in appropriate Laemmli buffer volumes to normalize for differences in OD_600_) were subjected to SDS-PAGE and visualized on a phosphorimager. Uncropped image shown in Supplementary Information Fig. 1.

An *in vitro* translation system was used to determine if the strong growth arrest phenotype characteristic of VapC-mt11 was a consequence of defective protein synthesis. Preincubation of the *in vitro* translation mix with VapC-mt11 before DHFR DNA template addition resulted in complete inhibition of synthesis relative to the control lane, which was not treated with recombinant VapC-mt11 (Fig. 2A). This translation defect was confirmed upon VapC-mt11 expression in *M. smegmatis* (Fig. 2B). Cells were radioactively labeled at intervals after VapC-mt11 induction to monitor new protein synthesis. Nearly complete shutdown of protein synthesis was observed 2 h after induction (the point where growth inhibition commences Fig. 1A) followed by sustained and complete inhibition of protein synthesis after 4 h of toxin induction. Therefore, the strong growth arrest phenotype characteristic of VapC-mt11 expression appears to be a consequence of complete translation inhibition in mycobacteria.

### VapC-mt11 is a tRNase that targets multiple synthetic *M. tuberculosis* tRNAs

**Figure 3:**
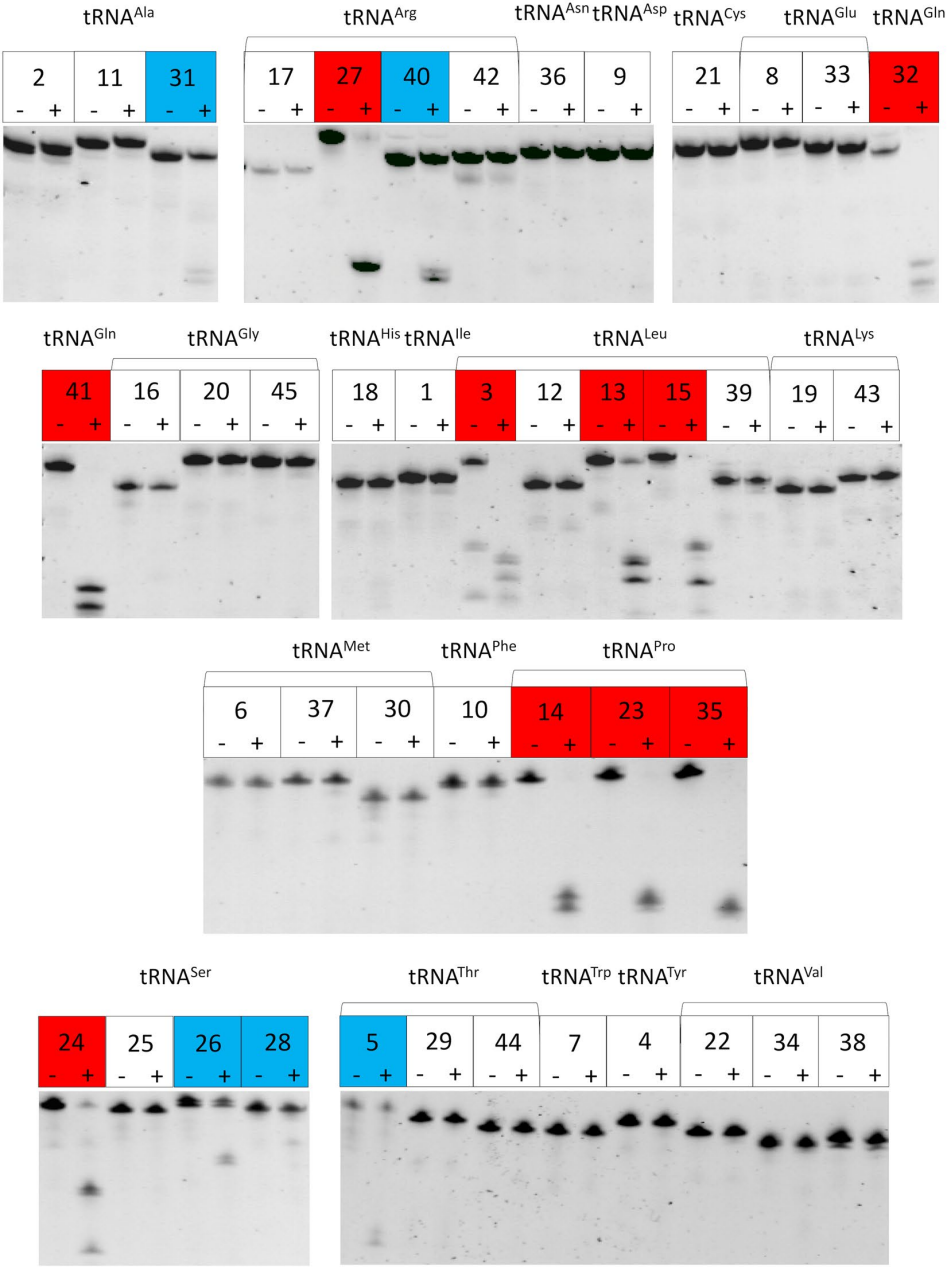
VapC-mt11 cleaves one third of *M. tuberculosis* tRNAs *in vitro*. tRNAs and their cleavage products visualized by SYBR Gold staining. 2 pmol of each of the 45 *in vitro* synthesized *M. tuberculosis* tRNAs was incubated with (+) or without (−) 10 pmol of recombinant VapC-mt11 for 3 h at 37 °C to ensure detection of both strong and weak tRNA cleavage reactions. Full length tRNAs that were cleaved to completion or near completion by VapC-mt11 shown in red; these tRNAs were also cleaved to the same extent shown when incubated for only 15 min at 37 °C. Weak VapC-mt11 tRNA targets shown in blue. tRNA numbering and anticodon sequences of each numbered tRNA from the Lowe lab genomic tRNA database http://gtrnadb.ucsc.edu^33^. All uncropped images shown in Supplementary Information Fig. 2.

Since there is precedent for VapC recognition of both tRNA and rRNA targets that is highly structure-dependent^8,14^, we assayed VapC-mt11 cleavage activity with each of these two intrinsically folded classes of RNAs. We first analyzed 23S and 16S rRNA from *E. coli* cells expressing VapC-mt11 and did not observe a decrease in the overall abundance of these rRNAs or the presence of any degradation products (data not shown). This suggested that VapC-mt11 likely targets tRNAs. Next, to determine which tRNAs were preferred substrates of VapC-mt11, each of the 45 tRNAs present in *M. tuberculosis* were synthesized *in vitro* and incubated with recombinant VapC-mt11 (Fig. 3). To our surprise, VapC-mt11 cleaved 15 tRNAs, one third of the 45 *M. tuberculosis* tRNAs. However, among these 15, ten were cleaved efficiently (eight to completion, two ~90% cleaved; shown in *red*, Fig. 3) while five exhibited only weak cleavage (*blue*, Fig. 3) The extent of cleavage did not change when assay times were increased (data not shown).

### VapC-mt11 tRNA recognition and cleavage is not modification dependent

**Figure 4:**
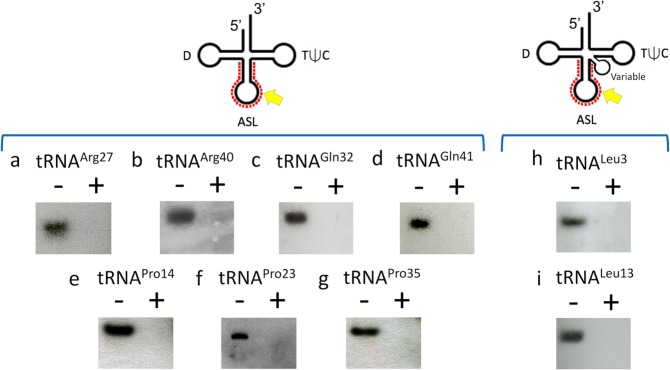
tRNA modifications are not required for VapC-mt11 target recognition *in vitro*. Total *M. tuberculosis* RNA was incubated with (+) or without (−) recombinant VapC-mt11. Northern analysis of (**a**) tRNA^Arg27^ (**b**) tRNA^Arg40^ (**c**) tRNA^Gln32^ (**d**) tRNA^Gln41^ (**e**) tRNA^Pro14^ (**f**) tRNA^Pro23^ (**g**) tRNA^Pro35^ (**h**) tRNA^Leu3^ (**i**) tRNA^Leu13^. The light band in the + lane of panel *b* is background, not uncleaved tRNA. Position of the oligonucleotides used are shown by the red dots on the tRNA diagrams above each bracketed group. Cleavage positions, yellow arrow. Oligonucleotides were designed to hybridize to the ASL to optimally differentiate between tRNA species. Therefore, cleavage products were generally not visible because the oligonucleotides could no longer hybridize to the cleaved ASL. Hybridization temperatures were optimized to preclude cross-hybridization to other tRNAs. tRNA numbering and anticodon sequences of each numbered tRNA from the Lowe lab genomic tRNA database http://gtrnadb.ucsc.edu^33^. All uncropped images shown in Supplementary Information Fig. 3.

**Figure 5:**
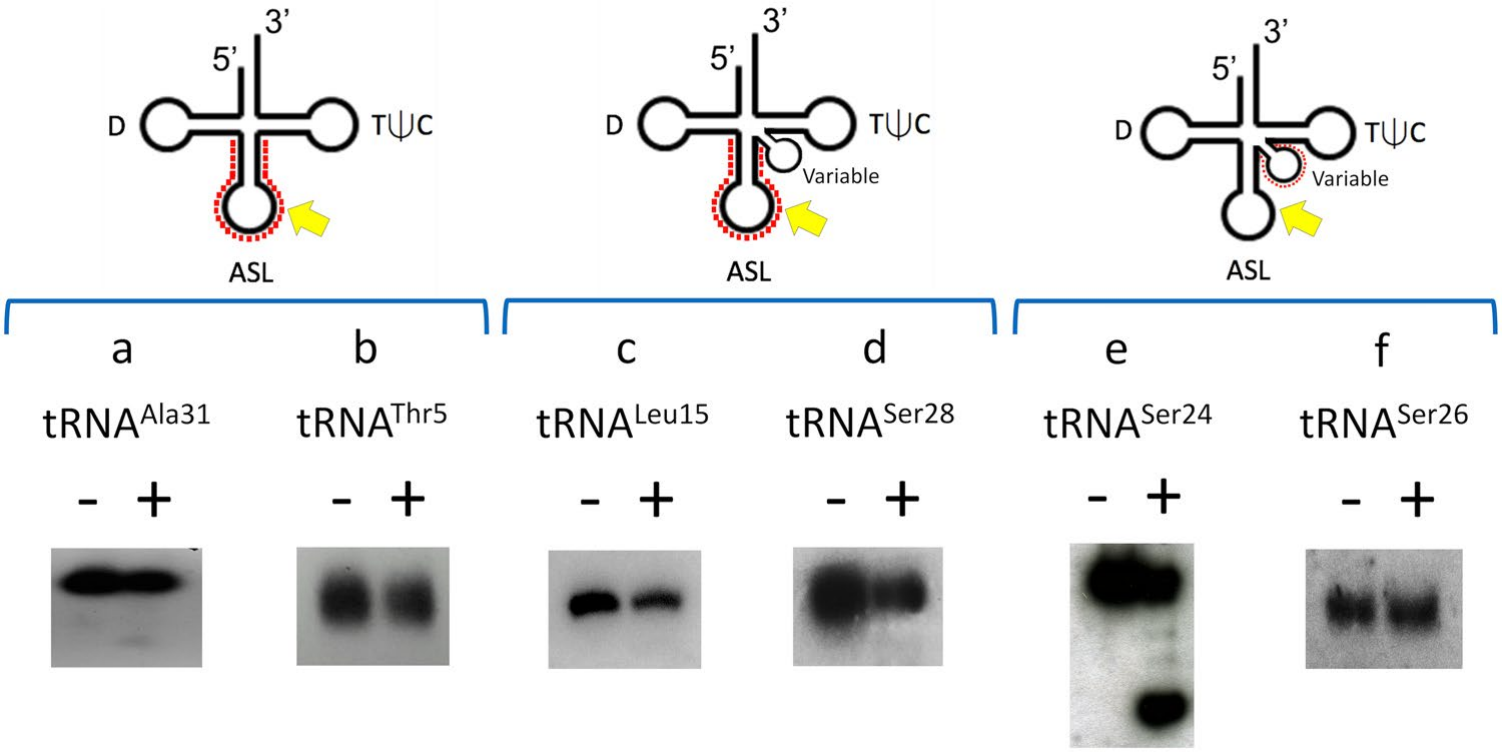
Weak or uncleaved *in vivo* synthesized tRNAs mirror the subset of weakly cleaved *in vitro* synthesized tRNAs. Total *M. tuberculosis* RNA was incubated with (+) or without (−) recombinant VapC-mt11. Northern analysis of (**a**) tRNA^Ala31^ (**b**) tRNA^Thr5^ (**c**) tRNA^Leu15^ (**d**) tRNA^Ser28^ (**e**) tRNA^Ser24^ (**f**) tRNA^Ser26^. Position of the oligonucleotides used are shown by the red dots on the tRNA diagrams above each bracketed group. Cleavage positions, yellow arrow. Oligonucleotides were designed to hybridize to the ASL to optimally differentiate between tRNA species. Therefore, cleavage products are not visible. Hybridization temperatures were optimized to preclude cross-hybridization to other tRNAs. tRNA numbering and anticodon sequences of each numbered tRNA from the Lowe lab genomic tRNA database http://gtrnadb.ucsc.edu^33^. All uncropped images shown in Supplementary Information Fig. 4.

To determine if posttranscriptional modifications present on tRNAs influenced cleavage, we used northern analysis to assess the cleavage efficiency of VapC-mt11 for all 15 tRNAs synthesized *in vitro* (Fig. 3) versus *in vivo* in *M. tuberculosis* cells (Figs 4 and 5). Generally, tRNA cleavage efficiency trends were in agreement. The *in vivo* counterparts for eight of the ten *in vitro* tRNAs that were cleaved to ~90–100% completion (Fig. 3, *red*) were also completely cleaved (Fig. 4). The two exceptions, tRNA^Leu15^ and tRNA^Ser24^, were ~50% cleaved by VapC-mt11 when derived from Mtb cells (Fig. 5c,e, respectively). Four of the five very weakly cleaved *in vitro* synthesized tRNAs (*blue* tRNAs in Fig. 3) exhibited marginal or no detectable cleavage by northern analysis (Fig. 5). The exception was *in vivo* synthesized tRNA^Arg40^, which was fully cleaved by VapC-mt11 (Fig. 5b). Since cleavage efficiency was generally comparable between synthetic tRNA and its counterpart from *M. tuberculosis* cells, VapC-mt11 target recognition and cleavage did not appear to require tRNA modifications.

### VapC-mt11 generates tRNA halves upon cleavage after a GG sequence in the anticodon loop

**Figure 6:**
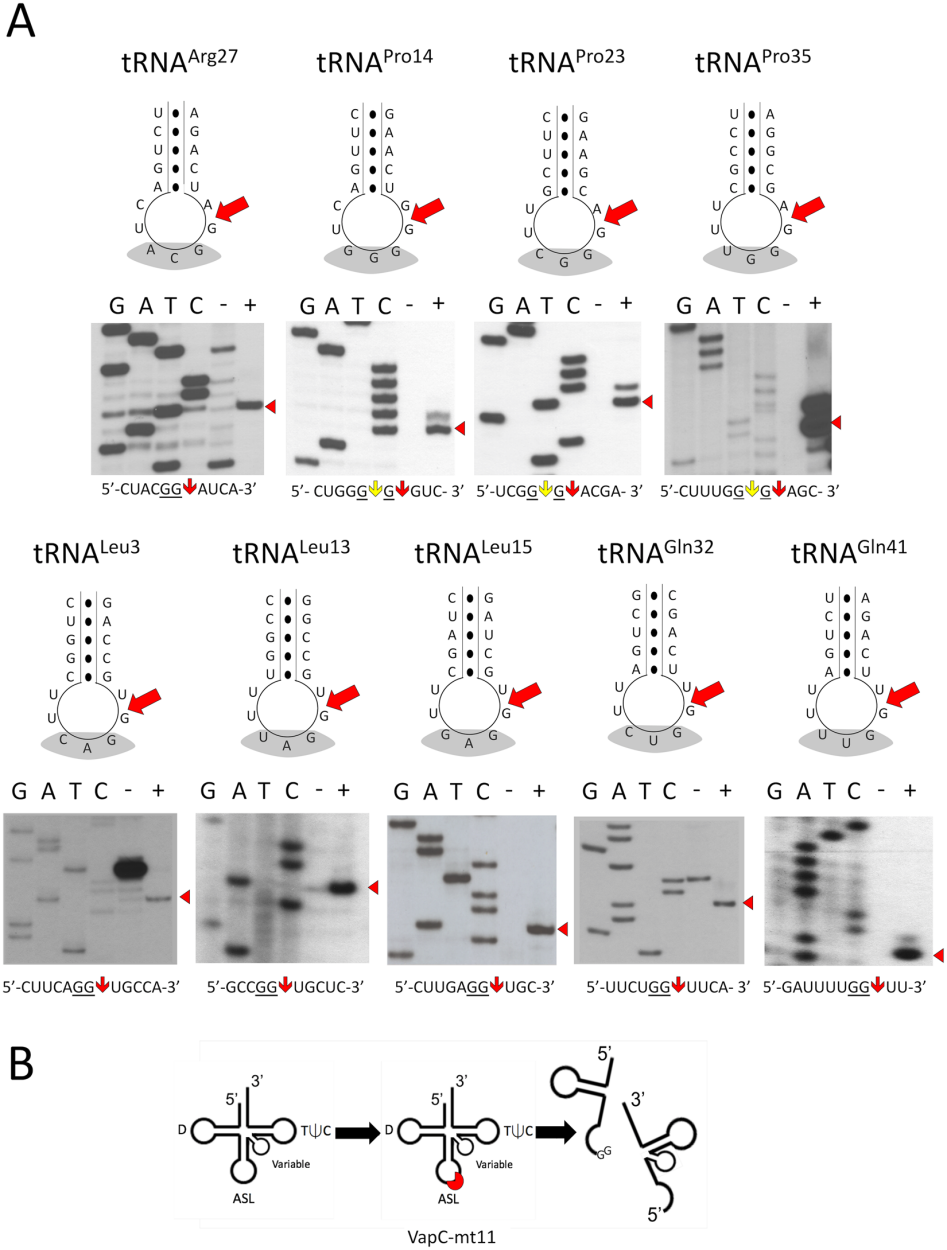
VapC-mt11 cleaves tRNAs within the ASL. (**A**) Primer extension analysis of *in vitro* synthesized *M. tuberculosis* tRNA^Arg27^, tRNA^Pro14^, tRNA^Pro23^, tRNA^Pro35^, tRNA^Leu3^, tRNA^Leu13^, tRNA^Leu15^, tRNA^Gln32^ and tRNA^Gln41^ treated with (+) or without (−) recombinant VapC-mt11. G, A, T, and C correspond to DNA-sequencing ladders using the same oligonucleotide as in the primer extension reactions for each tRNA. RNA sequence shown below the gels and major cleavage sites are indicated by the red arrow; alternate weak cleavage sites are indicated by yellow arrow for the three proline tRNAs. Major cleavage products in the gels are depicted by the red arrow head (right). The ASL sequence is illustrated above each tRNA sequencing gel, the anticodon (grey shading) and major cleavage position indicated by red arrow. Bands visible in (−) lanes of tRNA^Arg27^, tRNA^Leu3^ and tRNA^Gln32^ correspond to secondary structure. (**B**) Diagram of tRNA halves produced by VapC-mt11. tRNA numbering and anticodon sequences of each numbered tRNA from the Lowe lab genomic tRNA database http://gtrnadb.ucsc.edu^33^. All uncropped images shown in Supplementary Information Fig. 5 (tRNA^Arg27^, tRNA^Pro14^, tRNA^Pro23^, and tRNA^Pro35^) and Supplementary Information Fig. 6 (tRNA^Leu3^, tRNA^Leu13^, tRNA^Leu15^, tRNA^Gln32^, and tRNA^Gln41^).

We used primer extension analysis to map the VapC-mt11 cleavage sites for nine tRNAs that were efficiently cleaved *in vitro* (Fig. 6A), all but one of these (tRNA^Leu15^) were also 100% cleaved *in vivo*. In each case, three trends were observed. First, the primary cleavage site always occurred after a GG sequence (GG↓). Since we observed cleavage at GG ↓A, GG↓U and GG↓G sequences among these nine tRNAs, the base following cleavage site does not appear to contribute to target specificity. Yet, several GG-containing mRNAs were not cleaved by VapC-mt11. For example, *E. coli* OmpF and OmpC mRNAs contain 67 and 65 GG motifs, respectively, but were not cleaved by VapC-mt11 (data not shown). Likewise, as mentioned earlier, *E. coli* 23S and 16S rRNAs were not also cleaved, and they contain hundreds of GG motifs. Therefore, the presence of a GG RNA consensus sequence alone cannot serve as the sole determinant for tRNA target recognition. Second, all nine cleavage sites occurred at the same position in the anticodon loop, 3′ of the ribonucleotide that followed the anticodon. Third, VapC-mt11 always targeted the anticodon loop. This is consistent with our earlier observation for another VapC family member and a subsequent paper demonstrating that several *M. tuberculosis* VapC toxins target tRNAs for cleavage at their anticodon loop^7,8^. Therefore, VapC-mt11, and all *M. tuberculosis* VapC toxins known to cleave tRNA, result in the generation of tRNA halves (Fig. 6B).

### VapC-mt11 requires a GG sequence for tRNA recognition and accurate cleavage

**Figure 7:**
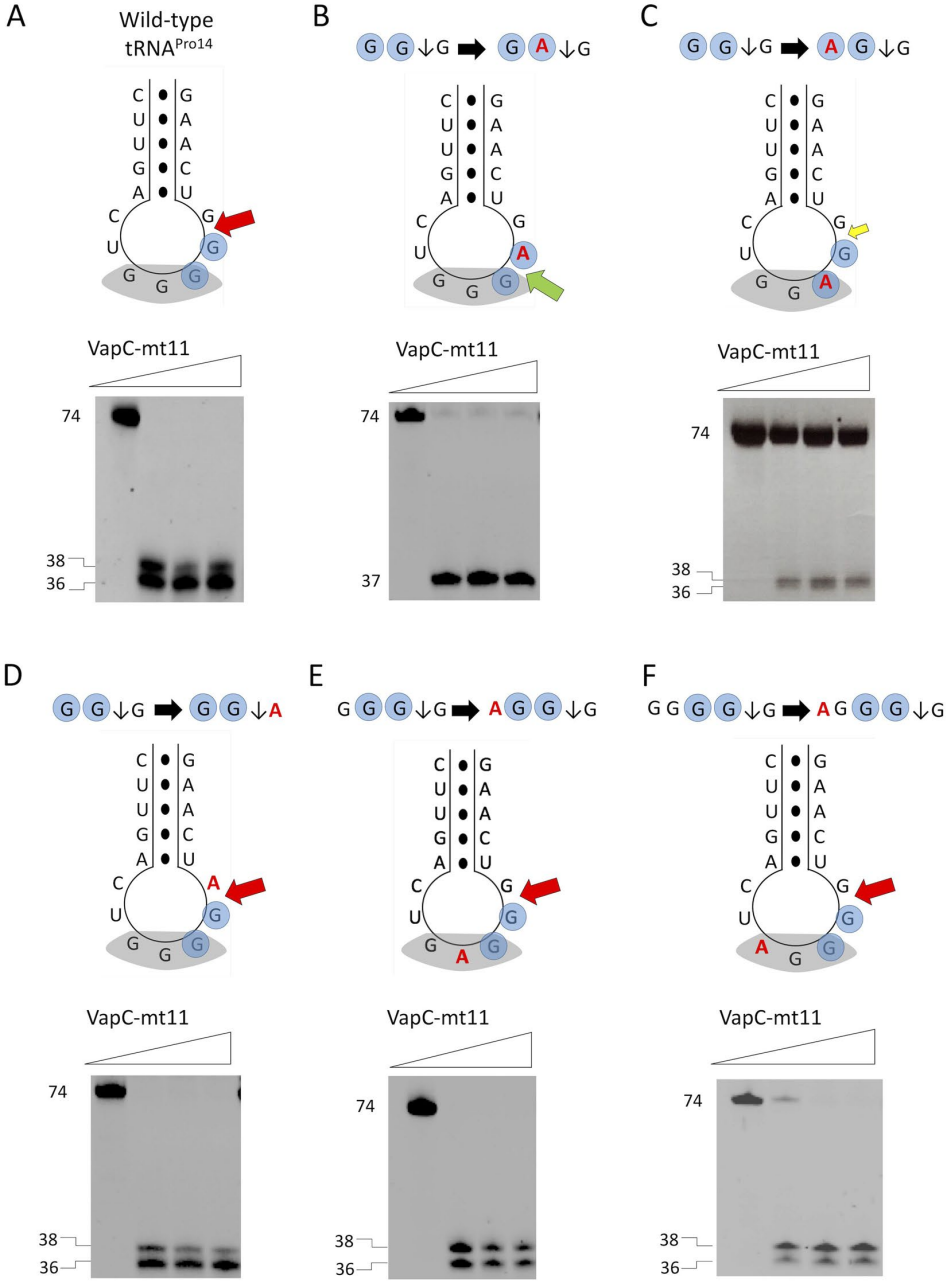
VapC-mt11 requires a GG sequence within the proper context. *In vitro* synthesized *M. tuberculosis* tRNA^Pro14^ mutants were incubated with increasing amounts of recombinant VapC-mt11 (ratios of toxin:tRNA were 0:1, 5:1, 10:1, 15:1) for 3 h at 37 °C. Sizes for full-length and cleavage products (left). Cleavage assays with *in vitro* synthesized wild-type tRNA^Pro14^ (**A**), tRNA^Pro14^ point mutation (G −> A) of each G residue of the consensus GG sequence (blue shaded circles) mutants (**B**,**C**), tRNA^Pro14^ mutant with point mutation 3′ of cut site (**D**) and tRNA^Pro14^ mutants with point mutations within the anticodon sequence (**E**,**F**). Secondary structure of *M. tuberculosis* tRNA^Pro14^ wild-type or mutants shown above gels. Anticodon sequence (shaded grey), base pairing represented as black dots (●), mutated bases (red), consensus sequence (shaded blue circles), wild-type cleavage site (red arrow), alternate cleavage site (green arrow), weak cleavage at wild-type cleavage site (small yellow arrow). All uncropped images shown in Supplementary Information Fig. 7.

To pinpoint the determinants that contribute to toxin recognition and cleavage by VapC-mt11, we created a variety of mutants using one of the efficiently cleaved tRNAs, tRNA^Pro14^, as the test tRNA template (Fig. 7A). First, we interrogated the importance of the conserved GG sequence 5′ of the cleavage site. Mutation of the second G of the GG consensus to an A (GG→GA) resulted in the shifting of the cleavage site one nt 5′ from the major cleavage site (which also corresponded to the position of the minor cleavage site in wild-type tRNA^Pro14^, Fig. 7B). Next, we mutated the first G of the GG consensus to an A (GG→AG). In this case, we observed very inefficient cleavage, with only a small percentage of the tRNA^Pro14^ template cut by VapC-mt11 (Fig. 7C).

Since we observed cleavage at GG↓A, GG↓U and GG↓G sequences among the 10 preferred tRNA targets (Fig. 6A), the base following the GG consensus sequence did not appear to contribute to target specificity. In agreement with this prediction, cleavage was not affected when we mutated the base 3′ of the GG consensus in tRNA^Pro14^ (Fig. 7D).

Next, we individually mutated each of the two bases 5′ of the GG cleavage consensus sequence, which in each case alters the anticodon sequence (GGG↓G→ AGG↓G, Fig. 7E; GGGG↓G→ AGGG↓G, Fig. 7F). Neither mutation altered the cleavage of tRNA^Pro14^ by VapC-mt11. Therefore, the only sequence determinants for cleavage of tRNA^Pro14^ by VapC-mt11 were the two GG residues directly 5′ of the cleavage site.

### VapC-mt11 cleavage is both sequence- and structure-dependent

**Figure 8:**
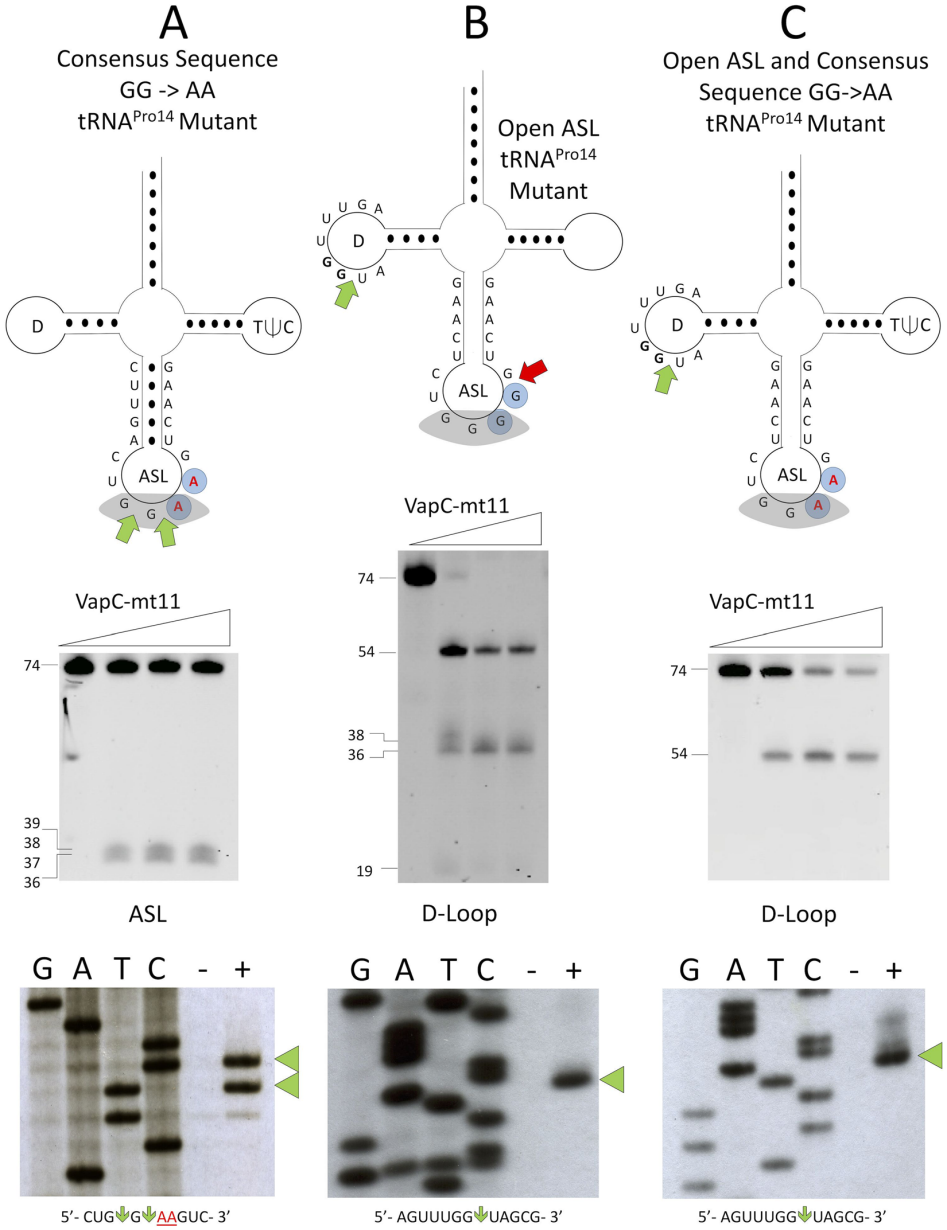
Sequence and structure influence the VapC-mt11 cleavage site. Cleavage assays of (**A**) tRNA^Pro14^ mutant with a GG consensus sequence replacement by AA, (**B**) an open stem loop mutant, and a (**C**) GG −> AA and open stem loop mutant. Full length and cleavage product sizes shown on the left. The small 19 nucleotide cleavage product ran off the gel in (**C**). Secondary structures of the *in vitro* synthesized mutants of tRNA^Pro14^ are depicted above cleavage reaction gels. Anticodon stem loop (ASL), anticodon sequence (shaded grey), base pairing represented as black dots (●), mutated bases (red), consensus sequence (blue shaded circles), weak cleavage at wild-type cleavage site (small yellow arrow), and alternate cleavage site (green arrow). Primer extension of these mutants shown below. G, A, T, and C correspond to DNA-sequencing ladders using the same oligonucleotide as in the primer extension reactions for each tRNA. Alternate cleavage sites are indicated by the green arrow head to the right of the primer extension gel images. RNA sequence corresponding to the alternate cleavage sites (green arrow) within the (**A**) ASL or (**B**,**C**) D-loop are shown below each primer extension gel image, mutations indicated by underlined red text. All uncropped images shown in Supplementary Information Fig. 8.

The importance of a GG sequence for VapC-mt11 cleavage is underscored when both consensus GG residues were simultaneously mutated to AA (GG→AA, Fig. 8A). In this case, we observed a two base 5′ shift in the cleavage site to the only other GG sequence remaining in the anticodon loop, suggesting that *in vitro* VapC-mt11 will seek out a GG in proximity when the native sequence is no longer in place.

Since we established that the presence of the GG sequence alone could not account for the specificity of VapC-mt11 for tRNAs, we first altered the structure of the anticodon stem loop (ASL) by abolishing stem formation (Fig. 8B). This relatively severe structural change resulted in an equally dramatic shift in the cleavage site. This open ASL mutant primarily shifted the cleavage site from the GG in the anticodon loop to a GG sequence in the D-loop of tRNA^Pro14^ (Fig. 8B, *green arrow*); only marginal cleavage at the native GG site was detected (Fig. 8B*, small yellow arrow*).

Finally, when we combined the open ASL mutation (Fig. 8B) with the consensus GG→AA mutation (Fig. 8A), we observed a complete shift of the cleavage site to GG↓ in the D-loop (Fig. 8C). Apparently, a GG sequence in the loop of an intact stem-loop (much like the original site harbored in the loop of the ASL) was a preferred alternate substrate over other spatially distinct GG motifs present in tRNA^Pro14^. More specifically, a second GG located in a single-stranded region between the ASL and TψC loop of tRNA^Pro14^ was not cut in this mutant. Alternatively, the selection of a new cleavage site in this mutant may simply be dictated by toxin accessibility as the D-loop would be predicted to be more surface exposed than the other GG site tucked between two stem loops.

In summary, VapC-mt11 requires the GG consensus sequence to be in the proper structural context within the anticodon loop for tRNA cleavage *in vitro*.

### VapC-mt11 cleaves only two GG consensus-containing tRNA targets when expressed in its natural *M. tuberculosis* host

We sought to understand why our *in vitro* cleavage experiments using either synthetic tRNAs or those isolated from *M. tuberculosis* (with modifications) identified numerous VapC-mt11 cleaved tRNAs compared to three identified by Gerdes and colleagues in a screen for *M. tuberculosis* VapC RNA targets expressed in another mycobacteria, *M. smegmatis*^7^.

**Figure 9:**
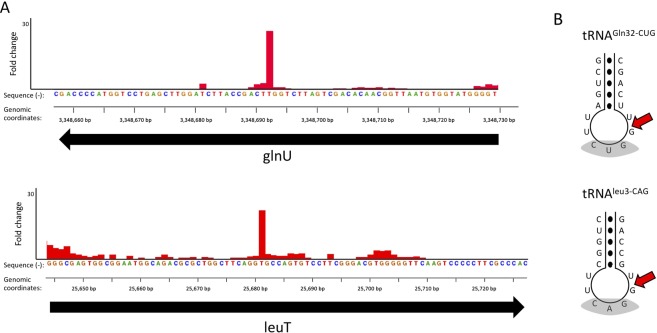
VapC-mt11 cleaves only two tRNAs containing the GG cleavage consensus sequence *in vivo*. (**A**) Histogram representing the ratio of cleavage by VapC-mt11 identified using 5′ RNA-seq at each nucleotide within the *glnU* gene (encoding tRNA^Gln32-CUG^) and LeuT gene (encoding tRNA^Leu3-CAG^) in *M. tuberculosis* after 24 hours of toxin induction. Genomic positions and the negative strand sequence for *glnU* and positive strand sequence for *leuT* are shown. (**B**) ASL sequence of the two *in vivo* VapC-mt11 tRNA targets, with cleavage site following the conserved GG consensus sequence indicated (red arrow).

We expressed VapC-mt11 in *M. tuberculosis* and performed a specialized RNA-seq method, 5′ RNA-seq, developed in our laboratory to identify RNA targets of endoribonuclease toxins^16^. 5′ RNA-seq methodology enables global analysis of specific populations of RNA transcripts based on the modification at their 5′ end^16^. VapC family members as well as other RNases have a hydroxyl group (5′-OH) at their 5′ ends^8,17^. Therefore, we analyzed only those transcripts carrying a 5′-OH in VapC-mt11 expressing *M. tuberculosis* cells compared to an uninduced control. The resulting dataset revealed both the RNA target and the position of toxin cleavage within these RNAs. Only two tRNAs were identified as VapC-mt11 targets, tRNA^Gln32-CUG^ and tRNA^Leu3-CAG^ (Fig. 9A). Both of these tRNAs were among the ten that were cleaved to completion *in vitro* (Fig. 3). In concordance with the requirements for substrate recognition and specificity we documented *in vitro*, both tRNA^Gln32-CUG^ and tRNA^Leu3-CAG^, were cleaved within their ASL and immediately after the GG consensus sequence to generate stable tRNA halves (Fig. 9B). Finally, 5′ RNA-seq did not identify any other class of RNA—mRNA, rRNA or other small stable noncoding RNA—that was directly cleaved by VapC-mt11.

## Discussion

Here we showed that a single toxin is capable of recognizing and cleaving nine *M. tuberculosis* tRNAs, one-fifth of the 45 distinct tRNA species present in this pathogen *in vitro* (Figs 3 and 4). The broad scope of targets identified *in vitro* seemed consistent with the growth and translation phenotypes observed. Expression of VapC-mt11 dramatically impaired *M. tuberculosis* growth (Fig. 1B) and precluded recovery of viable cells (Fig. 1C). Growth in *M. smegmatis* was also arrested ~2 h postinduction and beyond (Fig. 1A), concomitant with a virtually complete shutdown of new protein synthesis (Fig. 2A).

A common feature among these tRNA targets was sequence- (GG↓) and context-specific (anticodon loop of an intact ASL) cleavage. In fact, the requirement for proper structural context is emerging as a general feature of tRNA-cleaving *M. tuberculosis* toxins^8,18^. However, RNA target selection by VapC-mt11 in *M. tuberculosis* is dictated by more than the enzymatic properties of this toxin.

In its natural host VapC-mt11 was far more discriminating, cleaving only tRNA^Gln32-CUG^ and tRNA^Leu3-CAG^. This much higher level of target discrimination *in vivo* may be due to stress-specific modifications on the tRNAs or the binding of an accessory factor that alters enzyme specificity. However, evidence only exists to support the former explanation. Although we did not detect any difference in cleavage for synthetic tRNA^Gln32-CUG^ and tRNA^Leu3-CAG^ versus those derived from unstressed *M. tuberculosis* cells, newly added or newly removed post-transcriptional modifications to the tRNA that prevent cleavage of the other seven tRNAs *in vivo* might occur only after a specific stress and/or upon expression of the toxin. Although modifications on *M. tuberculosis* tRNAs have not been characterized in any detail, the ASL of tRNAs is generally a modification hotspot that influences tRNA structure and thermostability^19^. If the changes we observed in specificity are indeed attributable to modifications, the VapC-mt11 target recognition seen *in vivo* cannot be accurately recapitulated *in vitro* until the exact location and nature of the chemical changes on the entire *M. tuberculosis* tRNA population is elucidated.

The toxin components of Type II TA systems are characteristically activated in response to stress^3^. In *M. tuberculosis*, certain TA toxin transcripts are upregulated when cells are exposed to stresses relevant to latent tuberculosis infection—nutrient limitation^20–24^, hypoxia^4,24,25^, macrophage infection^4,26–28^ or antibiotic treatment^23,24,29–31^. However, there is no definitively confirmed stress trigger for any *M. tuberculosis* TA toxin linking upregulation of toxin mRNA to increased enzymatic activity of the toxin protein. By analogy, TA toxin expression may underlie the observation that hypoxia regulates tRNA modifications in the *M. tuberculosis* relative *M. bovis* BCG^32^.

There are five Leu tRNAs in *M. tuberculosis* to service the six Leu codons. tRNA^Leu3-CAG^ (depleted by VapC-mt11) services the most abundant of the six Leu codons, CUG. The Leu CUG codon is also one of the three most frequently represented codons in the *M. tuberculosis* transcriptome (50.4/1000 codons; equal to Gly GGC but less than Ala GCC at 59.8/1000 codons). The other four Leu tRNAs, are spared but may not be able to fully compensate for loss of tRNA^Leu3-CAG^.

There are two Gln tRNAs in *M. tuberculosis* to service the two Gln codons, CAG and CAA. tRNA^Gln32-CUG^ (depleted by VapC-mt11) services the CAG codon (22.8/1000 codons). If the 5′ anticodon C for tRNA^Gln32-CUG^ is modified, it may also service the CAA Gln codon (8.1/1000 codons). The second Gln tRNA (spared by VapC-mt11) services both Gln CAG and CAA codons.

Gerdes and colleagues identified RNA targets for several *M. tuberculosis* VapC toxins using a genome-scale toxin-RNA interaction screen in the rapidly growing, nonpathogenic *M. smegmatis*^7^. This screen identified three VapC-mt11-interacting *M. smegmatis* tRNAs*—*two isoacceptors of leucine, and one of glutamine. These three *M. smegmatis* tRNAs are designated tRNA^Leu3-CAG^, tRNA^Leu13-GAG^, and tRNA^Gln10-CUG^ and are orthologs of *M. tuber-culosis* tRNA^Leu3-CAG^, tRNA^Leu15-GAG^ and tRNA^Gln32-CUG^, respectively. Of the three *M. smegmatis* tRNAs, only tRNA^Leu3-CAG^ was significantly cleaved *in vivo* upon ectopic expression of VapC-mt11 in this surrogate host. Thus, only tRNA^Leu3-CAG^ in both *M. smegmatis* and *M. tuberculosis* was subjected to more detailed study. More specifically, (1) the sites of VapC-mt11 cleavage of *M. smegmatis* tRNA^Leu3-CAG^ *in vitro* were identified by primer extension and (2) relatively efficient *in vitro* cleavage of *M. tuberculosis* tRNA^Leu3-CAG^ was demonstrated^7^. Therefore, their data focused on tRNA^Leu3-CAG^ as the likely preferred RNA target for *M. tuberculosis* VapC-mt11. Our 5′ RNA-seq approach has three advantages over this interaction screen as well as *in vitro* cleavage assays similar to those presented in Figs 3 and 4. First, by focusing on only 5′-OH transcripts we are identifying transcripts that have been explicitly altered by the enzymatic activity of the toxin, not simply a cross-linkable RNA-protein interaction. Second, this method enables both the detection of the RNA target(s) as well as the exact position of cleavage without needing to perform laborious primer extension experiments. Third, enzyme activity occurs under physiological conditions (i.e., an *M. tuberculosis* host) to best approximate toxin activity during infection.

Recently, Deep *et al*. reported a high resolution x-ray crystal structure of VapBC-mt11 along with physiological studies of VapC-mt11^9^. VapC-mt11 overexpression in *M. tuberculosis* followed by RNA-seq led to an altered transcriptome that mirrored those of *M. tuberculosis* cells undergoing the enduring hypoxic response as well as cells in a nonreplicating persistent state^9^. Yet paradoxically, deletion of the *vapBC11* locus resulted in decreased recovery of viable cells only upon exposure to oxidative stress. Other stress conditions tested—including nonreplicating persistence, hypoxia using the Wayne model, nitrosative stress, nutritional stress, macrophage infection, and antibiotic treatment—did not alter cell recovery^9^. Finally, although not essential for cell survival, the *vapBC11* locus is required for establishment of an *M. tuberculosis* infection in the guinea pig model^9^.

In summary, our results reveal that analysis of toxin cleavage targets is most accurate in the *in vivo* setting in which the toxin is active, i.e. within the *M. tuberculosis* cell. Although *in vitro* cleavage methods are useful for identification of consensus sequences and structural features required for toxin cleavage, they have inherent limitations that may prelude accurate identification of the true toxin targets *in vivo*. Likewise, while ectopic toxin expression in alternate hosts followed by 5′ RNA-seq can provide useful clues to RNA targets or general RNA class favored by the toxin, the results will likely differ from that performed in the true host because of the inherent variability between transcriptomes and the presence and position of RNA modifications. Finally, expression of toxins in their natural host not only identifies the precise RNA target(s), it provides more physiologically relevant clues about the impact of toxin activity on discrete pathways and biological processes.

## Methods

### Strains, Plasmids and Reagents

The *E. coli* strain BL21(DE3) (F- *omp*T *hsd*S_β_(r_β_-m_β_) *dcm gal* (DE3) *ton*A) (Novagen) was used for all protein expression. *E. coli* K-12 Mach1 T1 cells (Δ*rec*A1398 *end*A1 *ton*A Φ80Δ*lac*M15 Δ*lac*X74 *hsd*R(r_k_^+^m_k_^+^); Invitrogen) were used for all cloning experiments. *M. smegmatis* mc^2^155 and *M. tuberculosis* H37Rv strains were both used for growth profiles. Experiments involving the virulent H37Rv strains of *M. tuberculosis* were conducted in a BSL-3 laboratory following institutionally approved protocols. *M. tuberculosis* H37Rv was also used for the plating efficiency assay to determine recovery of viable bacteria upon VapC-mt11 expression. *M. smegmatis* mc^2^155 was used for metabolic labeling. The vapC-mt11 (Rv1561; “mt” refers to *M. tuberculosis*) gene was cloned using *M. tuberculosis* H37Rv genomic DNA. The DNA sequences of PCR fragments used for cloning were confirmed by automated DNA sequence analysis. The VapC-mt11 coding region was cloned into the pET28a expression vector (EMD Millipore) and the arabinose inducible pBAD33 plasmid (ATCC) after the addition of 5′ NdeI and 3′ BamHI restriction enzyme sites by PCR. For expression in *Mycobacteria*, the VapC-mt11 coding region with PCR generated 5′ ClaI and 3′SalI restriction sites was cloned into the corresponding sites in the anhydrotetracycline (ATc) inducible vector pMC1s^15^.

### Growth Assays in Mycobacteria

*M. smegmatis* mc^2^155 was transformed by electroporation with 0.5–1 μg of pMC1s-*vapC-mt11* DNA. Cultures were inoculated with single colonies, grown at 37 °C in 7H9-TW80-AND medium containing 25 μg/ml kanamycin and induced at an OD_600_ of 0.4 by addition of 200 ng/ml ATc. Cell pellets were collected from uninduced and induced samples at intervals up to 6 h. *M. tuberculosis* H37Rv was transformed by electroporation with 0.5 μg pMC1s-*vapC-mt11* DNA. Cultures were inoculated with single colonies, grown at 37 °C in 7H9-TW80-AND medium containing 30% spent medium and 25 μg/ml kanamycin. Upon inoculation, cultures were induced by 200 ng/ml ATc; additional ATc was added every other day to maintain the initial ATc concentration. Three independent experiments were performed and error bars used to represent the S.D. For plating efficiency assays, *M. tuberculosis* H37Rv VapC-mt11 clones were plated on 7H9-TW80-ADN agar plates containing 25 μg/ml kanamycin and with or without 500 ng/ml ATc. Colonies were counted 3-weeks after incubation at 37 °C.

### Purification of Recombinant VapC-mt11

*E. coli* BL21(DE3) cells transformed with the pET28a-*vapC-mt11* were grown in M9 minimal medium supplemented with 0.1% glycerol and 50 μg/ml kanamycin at 37 °C to exponential phase. Induction of the protein was achieved by adding 1 mM isopropyl 1-thio-D-galactopyranoside (IPTG). After 4 h, cells were harvested by centrifugation and resuspended in lysis buffer (50 mM NaH_2_PO_4_ (pH 8.0), 500 mM NaCl, 20 mM imidazole, 10 mM β-mercaptoethanol, 1 mM PMSF, 1 mg/ml lysozyme). Cell pellets were then lysed by sonication and lysates applied to a nickel-nitrilotriacetic acid (Ni-NTA) resin (Qiagen) to purify the protein as described previously^6^.

### *In vitro* Transcription of *M. tuberculosis* tRNAs

*M. tuberculosis* wild-type and mutant tRNAs were synthesized *in vitro* using synthetic DNA oligonucleotides containing the T7 RNA polymerase promoter and the 5′ end sequence of the tRNA of interest along with a second oligonucleotide with the 3′ end sequence of the tRNA. Oligonucleotides were designed with a region of overlap to serve as a template for PCR with Taq DNA polymerase to create a tDNA. The tDNA was gel extracted from a 2% agarose gel using the QIAquick Gel Extraction kit (Qiagen). 200 ng of the tDNA was used to transcribe the tRNAs of interest using the RiboMAX Large Scale RNA Production System (Promega) as recommended by the manufacturer. The tRNA transcription reactions were separated on a 9% polyacrylamide, 7 M urea gel and visualized by ethidium bromide staining. The product corresponding to the correct size was excised from the gel and incubated for 18 h at 37 °C in elution buffer (1 mM EDTA, 0.5 M ammonium acetate, 10 mM magnesium acetate, 0.1% SDS). The elutions were ethanol precipitated and resuspended in nuclease-free water.

### *In vitro* tRNA Cleavage Assay

The 45 *in vitro* transcribed *M. tuberculosis* tRNAs (2 pmol) were initially incubated with or without VapC-mt11 (10 pmol) for 3 hr at 37 °C in 10 mM HEPES pH 7.5, 15 mM KCl, 3 mM MgCl_2_, 10% glycerol. Mutants were also incubated with increasing amounts of the toxin (0, 10, 20 and 30 pmol) for 3 h at 37 °C. Samples were analyzed in 7 M Urea, 9% polyacrylamide gel and visualized by SYBR Gold (Invitrogen) staining.

### *In vitro M. tuberculosis* tRNA Primer Extension

2 pmol of the *in vitro* transcribed *M. tuberculosis* wild-type or mutant tRNAs were incubated with 10 pmol VapC-mt11 toxin or without toxin for 1 h at 37 °C. To detect cleavage products, the following oligonucleotides were 5′ labeled with [γ-^32^P] ATP (Perkin Elmer Life Sciences) using T4 polynucleotide kinase (NEB): tRNA^Ala31^ (5′GGA GCT AAG GGG ACT CGA ACC C 3′), tRNA^Arg27^ (5′GCG CCC GAA GAG ATT CGA ACT C3′), tRNA^Arg40^ (5′ CCG GCA GGA TTC GAA CCT GCG 3′), tRNA^Gln32^ (5′ CTG GGG TAC CAG GAC TCG A 3′), tRNA^Gln41^ (5′ TCC GTC GCC AGG ACT CGA ACC 3′), tRNA^Leu13^ (5′ GCG GGC GGA GGG ACT CGA ACC 3′), tRNA^Leu15^ (5′GGG ACT TGA ACC CCC ACG C 3′), tRNA^Pro14^ (5′ CGG GCT GAC AGG ATT TGA ACC TGC G 3′), tRNA^Pro23^ (5′ CGG GGT GGC GGG ATT TGA AC 3′), tRNA^Pro35^ (5′ CGG GGT GAC AGG ATT TGA ACC TG 3′), tRNA^Ser24^ (5′ GGA GGA TGC GGG ATT TGA ACC C 3′), tRNA^Ser26^ (5′ GAG GCG AGA GGA TTT GAA CCT CC 3′), tRNA^Ser28^ (5′ GGT GGC GGA GGG ATT TGA ACC CTC 3′), tRNA^Thr5^ (5′ GCC CCC TAA CGG AAT CGA ACC 3′). For tRNA^Pro14^ mutants, the tRNA^Pro14^ (5′ CGG GCT GAC AGG ATT TGA ACC TGC G 3′) was used. Labeled oligonucleotides were added to the reactions and incubated for 3 min at 95 °C and left to cool down to room temperature. Primer extension with Superscript III or IV (Invitrogen) was performed at >60 °C for 80 min. The DNA sequencing ladder was produced using the Sequenase version 2.0 DNA sequencing kit (USB) according to the manufacturer’s instructions. Loading buffer (95% formamide, 20 mM EDTA, 0.05% bromophenol blue and 0.05% xylene cyanol) was added to each sample followed by 15% Urea PAGE and autoradiography.

### tRNA Northern Analysis

In brief, total RNA from *M. tuberculosis H37Rv* was obtained from cells grown to exponential phase using TRIzol Reagent (Invitrogen). RNA was treated with TURBO DNase (Invitrogen). 1.5 μg of total RNA was incubated with 98 pmol of recombinant VapC-mt11 toxin for 3 h at 37 °C. Reactions were subjected to Urea-PAGE separation on a 9% polyacrylamide, 7 M urea gel and visualized by ethidium bromide staining and transferred to nitrocellulose. The following oligonucleotides were 5′ labeled with [γ-^32^P] ATP (Perkin Elmer Life Sciences) using T4 polynucleotide kinase (NEB): tRNA^Ala31^ (5′ CCA CAC TGC CAG TGT GGT GCG C 3′), tRNA^Arg27^ (5′ CCT TCT GAT CCG TAG TCA GAT GC 3′), tRNA^Arg40^ (5′ CTT CTG CTC CGG AGG CAG ACG 3′), tRNA^Gln32^ (5′ ATG GCT GAA CCA GAA TCA GCT GT 3′), tRNA^Gln41^ (5′ CTA TCT GAA CCA AAA TCA GAG GTG C 3′), tRNA^Leu3^ (5′GGA CAC TGG CAC CTG AAG CCA 3′), tRNA^Leu13^ (5′ GCA CCG GCA CCT AAA ACC GGC 3′), tRNA^Leu15^ (5′ TAG GGC ACT AGC ACC TCA AGC TAG CG 3′), tRNA^Pro14^ (5′GAC CAC TTG ACC CCC AGT CAA G′), tRNA^Pro23^ (5′GGC CTC TTC GTC CCG AAC GAA GC3′), tRNA^Pro35^ (5′ GGC CTT CCG CTC CCA AAG CGG AT 3′), tRNA^Ser24^ (5′AGG GCT GTT AAC CCA ACC CGC G 3′), tRNA^Ser26^ (5′ CCC TTG AAG GGG GAC AAC TCA TTA 3′), tRNA^Ser28^ (5′ CAC ACG CTT TCG AGG CGT GCT CC 3′), tRNA^Thr5^ (5′CCT TTT CCT TAC CAT GGA AAC G 3′). Hybridization temperatures used precluded cross-reactivity with other tRNAs.

### ***In vitro*** Translation Inhibition

To test whether the VapC-mt11 strong toxic phenotype was due to translation inhibition, we added recombinant VapC-mt11 to the PURExpress kit (New England Biolabs) at a concentration of 200 pmol. Production of the DHFR protein (~20 kDa) was assessed via [^35^S] Express Protein Labeling Mix (Perkin Elmer Life Sciences) incorporation. An equal volume of 2X Laemmli buffer (125 mM Tris (pH 6.8), 20% glycerol, 4% SDS, 0.01% bromophenol blue) was added to terminate the translation reaction. Samples were heated to 95 °C for 5 min prior to separation by 17.5% SDS-PAGE followed by autoradiography.

### Metabolic Labeling in *M. smegmatis*

Transformants were obtained as previously described. Individual colonies were grown at 37 °C in 7H9-TW80-AND medium containing 20 μg/ml kanamycin until OD_600_ 0.3–0.5. The culture was then split into equal portions, and 200 ng/ml anhydrotetracycline (ATc) was added to one portion. 1 ml aliquots were removed at 0, 0.5, 1, 2, 4 and 6 h post-induction and incubated with 37.5 μCi of [^35^S] Express Protein Labeling Mix (Perkin Elmer Life Sciences) at 37 °C for 20 min. Cell pellets were collected by centrifugation (13,200 rpm for 5 min) and resuspended in Laemmli buffer. To ensure normalization, the volume of Laemmli buffer added to each pellet was determined by multiplying the OD_600_ by 500. To lyse the *M. smegmatis* cells resuspended in Laemmli buffer, a 40% volume of acid washed glass beads (≤160 μm; Sigma) were added and the mixture vortexed for 5 min. Samples were heated to 95 °C for 5 min prior to 17.5% SDS-PAGE followed by autoradiography.

### 5’ RNA-seq

Total RNA was isolated from *M. tuberculosis* H37Rv harboring the plasmid pMC1s containing *vapC-mt11* were grown to an OD_600_ of 0.4 and split into uninduced and induced cultures (+200 ng/ml anhydrotetracycline). Samples were collected 8 h and 24 h post induction and pelleted by centrifugation. Preparation of 5′-dependent libraries and data analysis was performed as described in Schifano *et al*.^16^. Briefly, for sequencing of RNAs containing 5′ hydroxyl ends (5′-OH), total RNA samples were digested using 1 U of Terminator 5′-Phosphate-Dependent Exonuclease (Epicentre) to remove RNAs containing 5′-monophosphate (5′-P). After purification using RNA Clean & Concentrator™-5 (Zymo Research), 5′-OH ends were phosphorylated using 3 U of T4 PNK (New England Biolabs). The small RNA 5′ adapter (5′-GUUCAGAGUUCUACAGUCCGACGAUCNNNNNN-3′) was ligated to the phosphorylated 5′ ends using T4 RNA ligase 1 (New England Biolabs) at 16 °C overnight. Ligated RNAs were separated from free adapters on a 6% Urea-PAGE gel and isolated by gel excision. Subsequently, cDNA was generated using Superscript IV (ThermoFisher) with the primer 5′-GCCTTGGCACCCGAGAATTCCANNNNNNNNN-3′, and gel extracted selecting fragments from 80 to 500 nts. The cDNA libraries were subjected to 12 cycles of PCR amplification with Phusion HF DNA polymerase (ThermoFisher), using the oligonucleotides RP1 (5′-AATGATACGGCGACCACCGAGATCTACACGTTCAGAGTTCTACAGTCCGA-3′) and RPIX (5′-CAAGCAGAAGACGGCATACGAGATNNNNNNGTGACTGGAGTTCCTTGGCACCCGAGAATTCCA-3′), where underlined N’s represent the library-specific Illumina barcodes. After 10% PAGE, amplified DNA between the sizes 150 bp and 450 bp was isolated by gel excision and sequenced in an Illumina NextSeq. 500 platform.

After trimming the adapter sequences from the resulting FASTQ files, the sequences were trimmed to 20 nts (discarding shorter sequences) and aligned to the *M. tuberculosis* H37Rv genome (Genbank accession: AL123456) using Bowtie 1.2.0, not allowing mismatches. For each nucleotide in the genome, we calculated the number of reads that started at that position (i.e. the number of RNA molecules that had their 5′ OH end starting at each nucleotide). Read counts were normalized to sequencing depth and expressed as “reads per million of mapped reads” (rpm). The ratio of counts between induced and uninduced was calculated. Positions that had 0 counts in the uninduced library were adjusted to a pseudo-count of 0.5. We only considered reads that had at least 5 rpm for mRNAs and 50 rpm for tRNAs in the induced sample and a ratio of at least 10. Among the three tRNAs that met these criteria—tRNA^Gln32-CUG^, tRNA^Leu3-CAG^ and tRNA^Glu8-UUC^—only two were counted as legitimate primary VapC-mt11 targets (tRNA^Gln32-CUG^ and tRNA^Leu3-CAG^) because they had a GG cleavage consensus sequence immediately before the cleavage site. tRNA numbering based on the Lowe lab genomic tRNA database http://gtrnadb.ucsc.edu^33^. The sequencing datasets generated in this study were deposited in the NCBI Sequence Read Archive (Submission ID: PRJNA509278).

## Supplementary information

Supplementary Information
