# Supplementary material for: Accurate target identification for Mycobacterium tuberculosis endoribonuclease toxins requires expression in their native host
Source: Sci Rep. 2019 Apr 11;9:5949. doi: 10.1038/s41598-019-41548-9 (PMC6459853; doi:10.1038/s41598-019-41548-9)
Supplement: Supplementary file 1 — Supplementary Information [file 41598_2019_41548_MOESM1_ESM.pdf]

Supplementary Information

Accurate target identification for *Mycobacterium tuberculosis* endoribonuclease toxins requires expression in their native host

Melvilí Cintrón[1], Ju-Mei Zeng[†2], Valdir C. Barth[†1], Jonathan W. Cruz[1], Robert N. Husson[2], and Nancy A. Woychik[1]

[1] Department of Biochemistry and Molecular Biology, Rutgers University, Robert Wood Johnson Medical School, Piscataway, NJ 08854; Member, Rutgers Cancer Institute of New Jersey and the
[2] Division of Infectious Diseases, Boston Children's Hospital/Harvard Medical School, Boston, MA, 02115

[†]These authors contributed equally

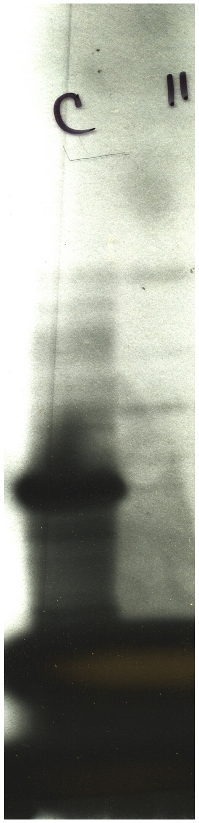

Figure 2A

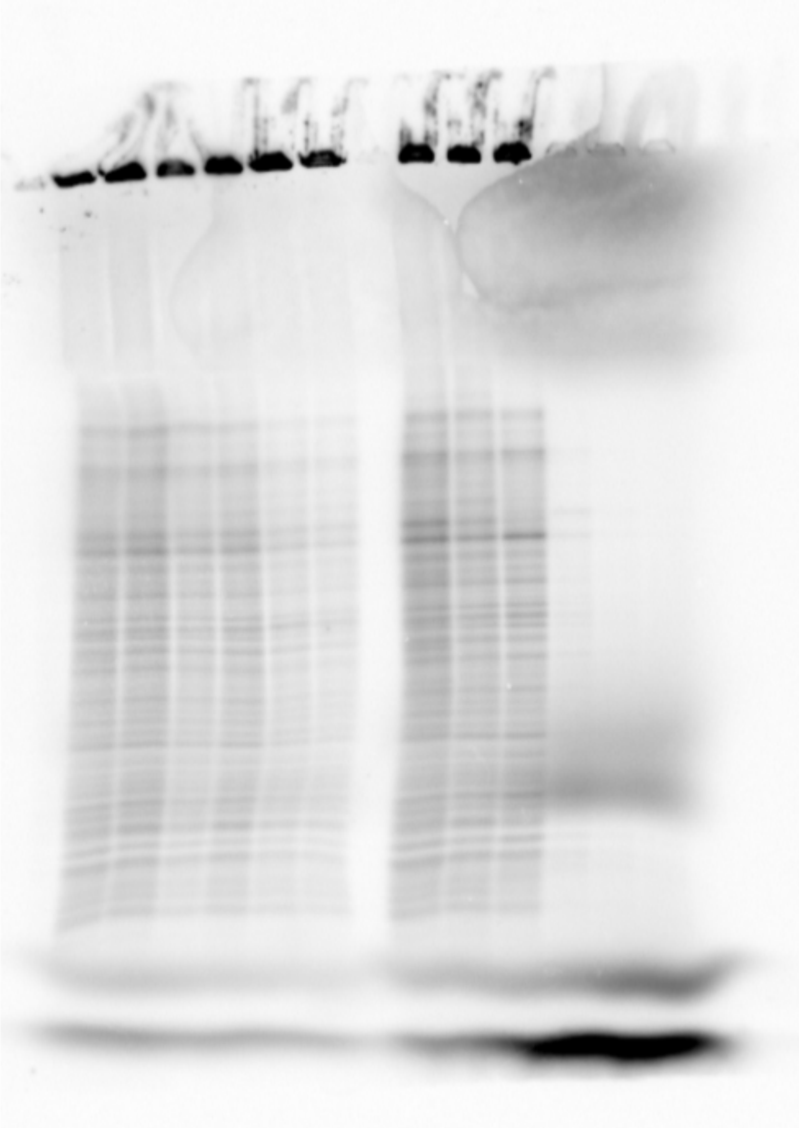

Figure 2B

<u>Supplementary Information Figure 1</u>: Uncropped Images from Figure 2

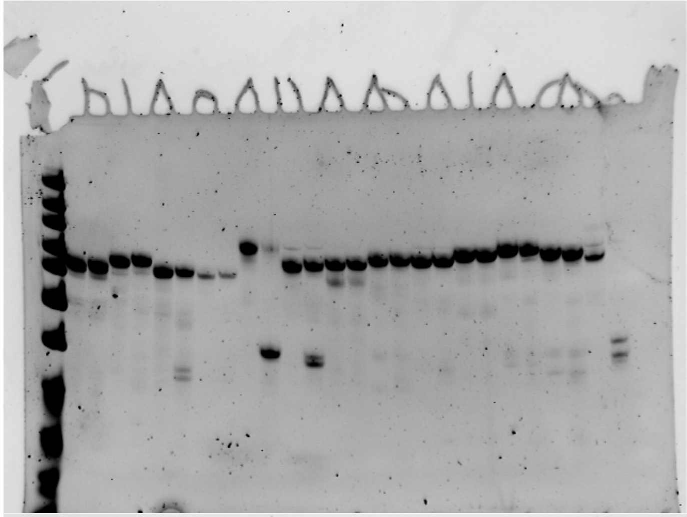

Top (first) panel Fig. 3

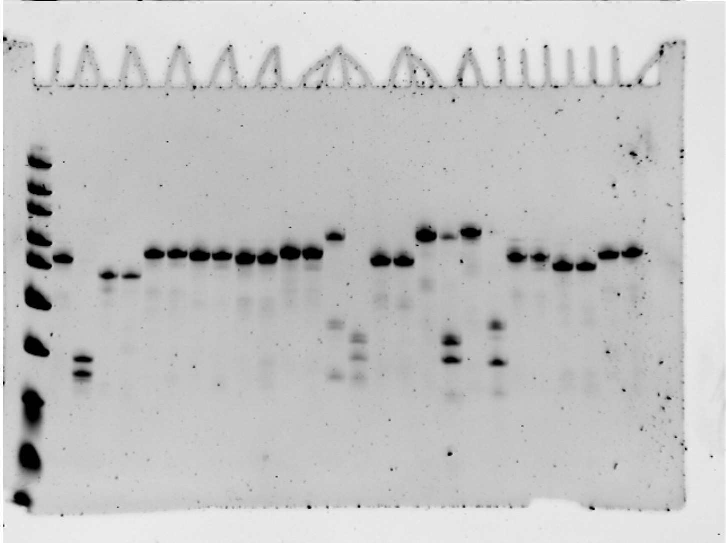

Second panel Fig. 3

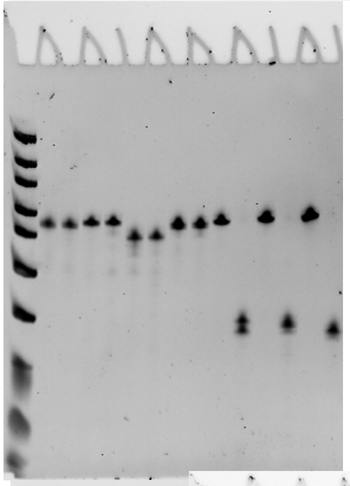

Third panel Fig. 3

<u>Supplementary Information Figure 2:</u>

Uncropped Images from Figure 3

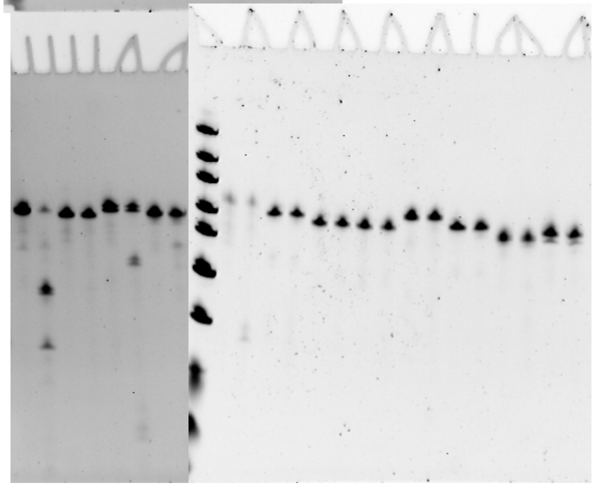

Forth panel Fig. 3

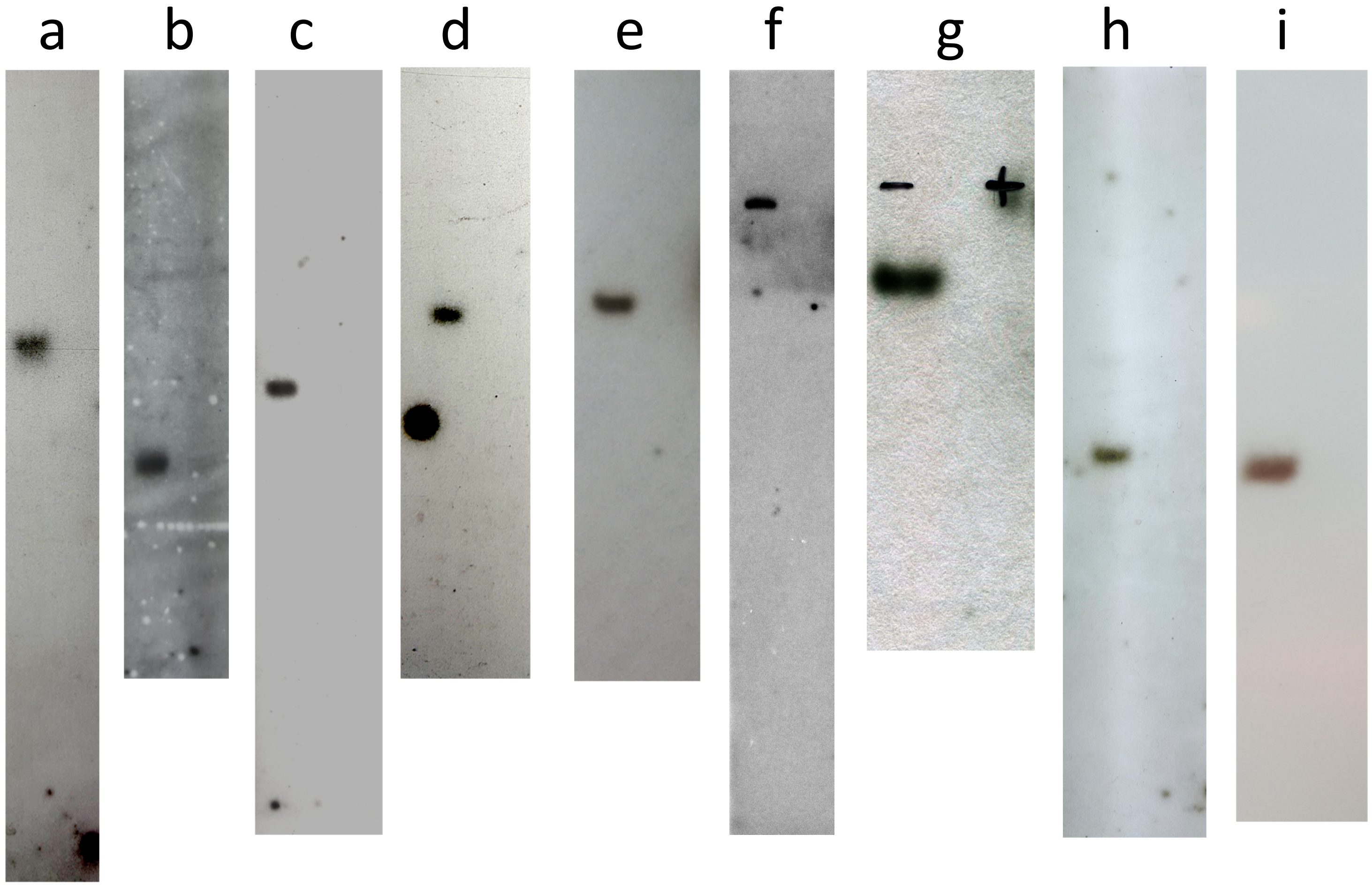

Supplementary Information Figure 3: Uncropped Images from Figure 4 panels a-i

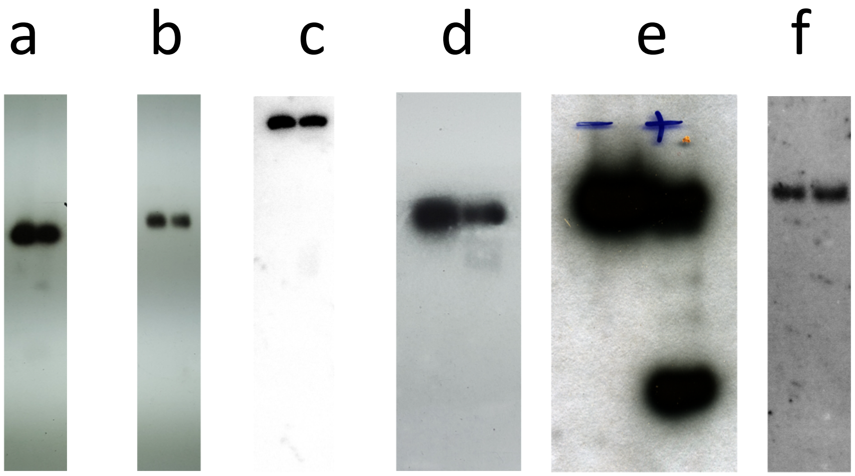

<u>Supplementary Information Figure 4:</u> Uncropped Images from Figure 5 panels a-f; some exposures shown here are even longer (darker) to ensure no cleavage products were missed

tRNA^{Arg27} tRNA^{Pro14} tRNA^{Pro23} tRNA^{Pro35}

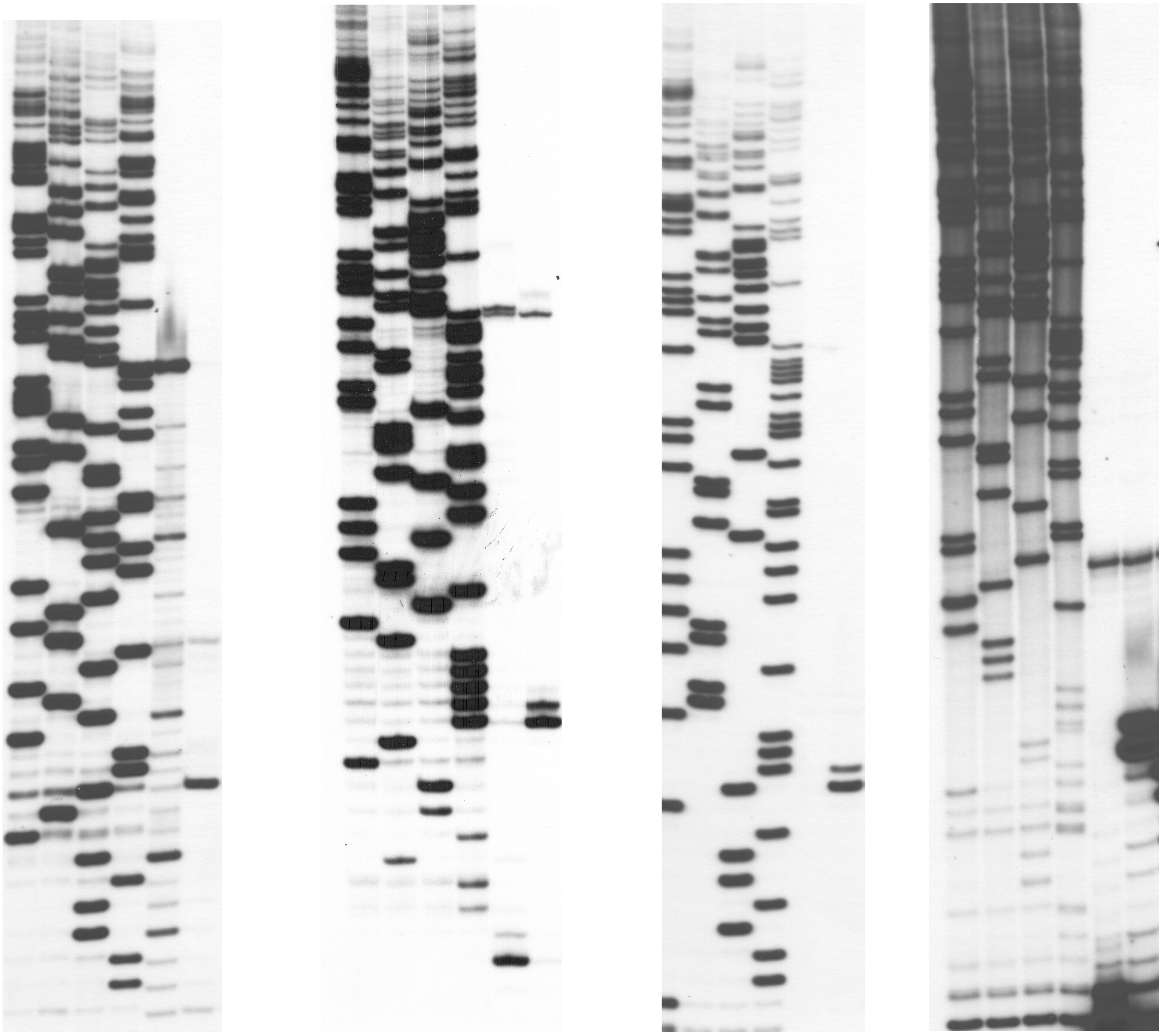

Supplementary Information Figure 5: Uncropped Images from top half of Figure 6

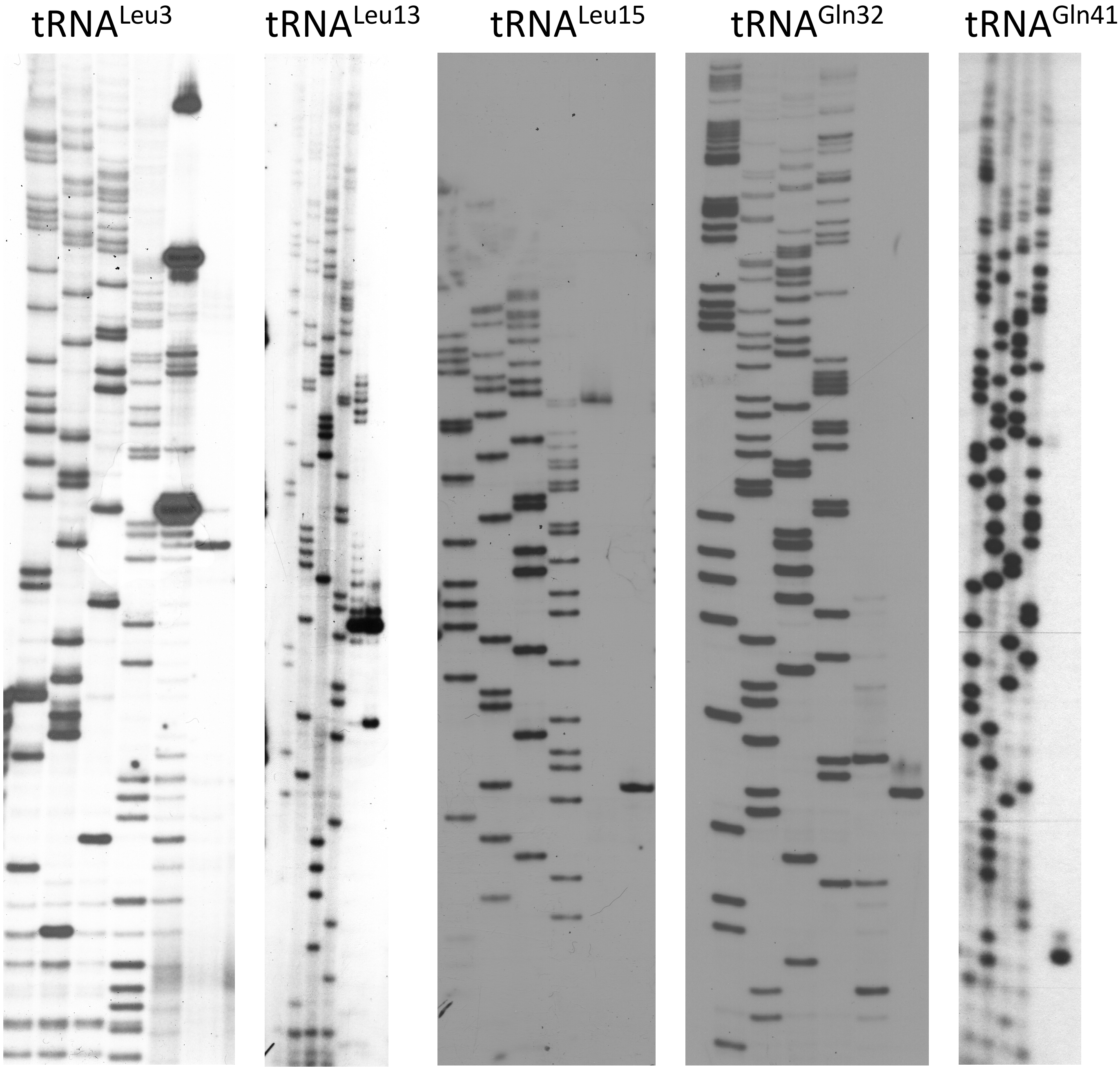

Supplementary Information Figure 6: Uncropped Images from bottom half of Fig. 6

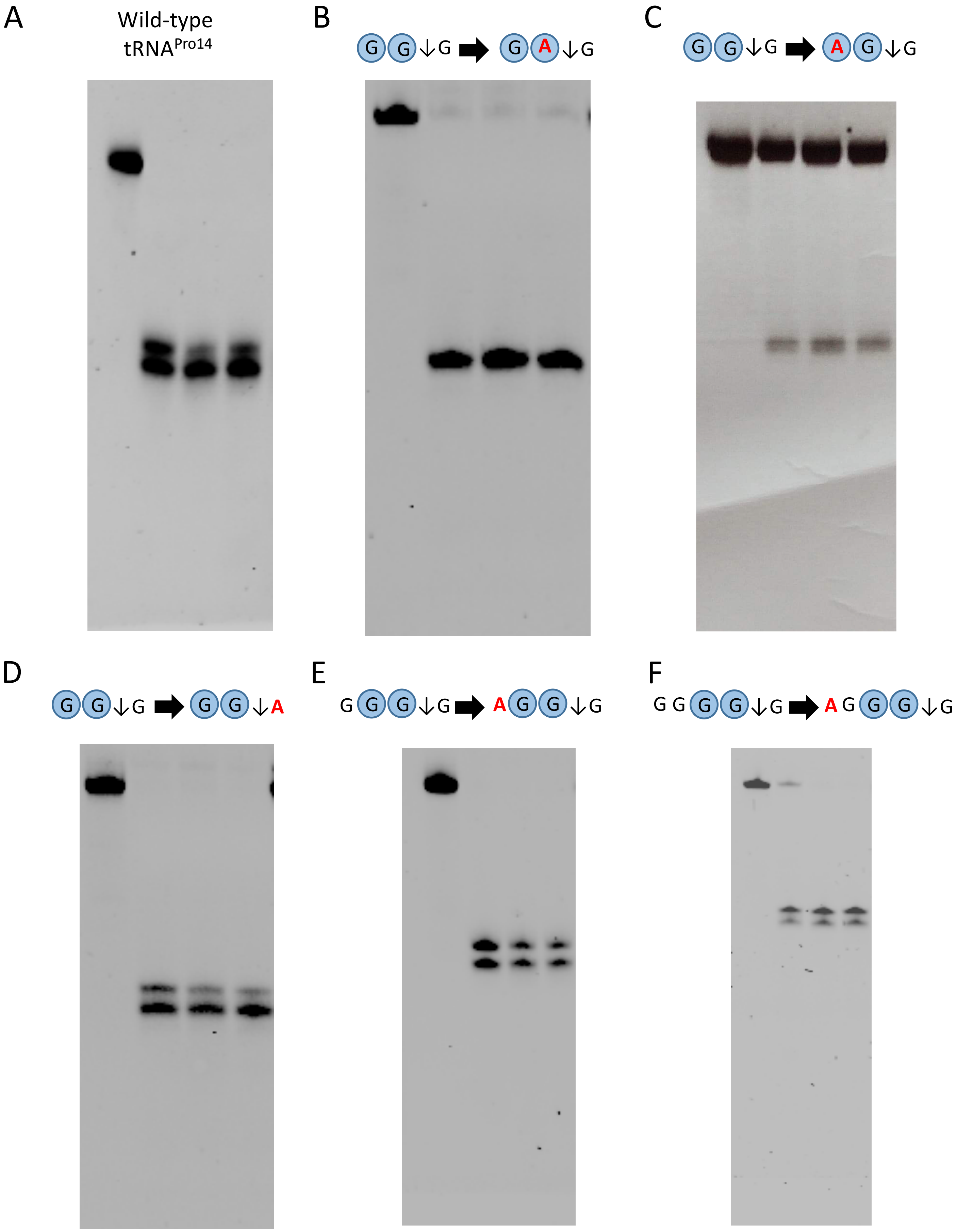

Supplementary Information Figure 7: Uncropped Images from Figure 7

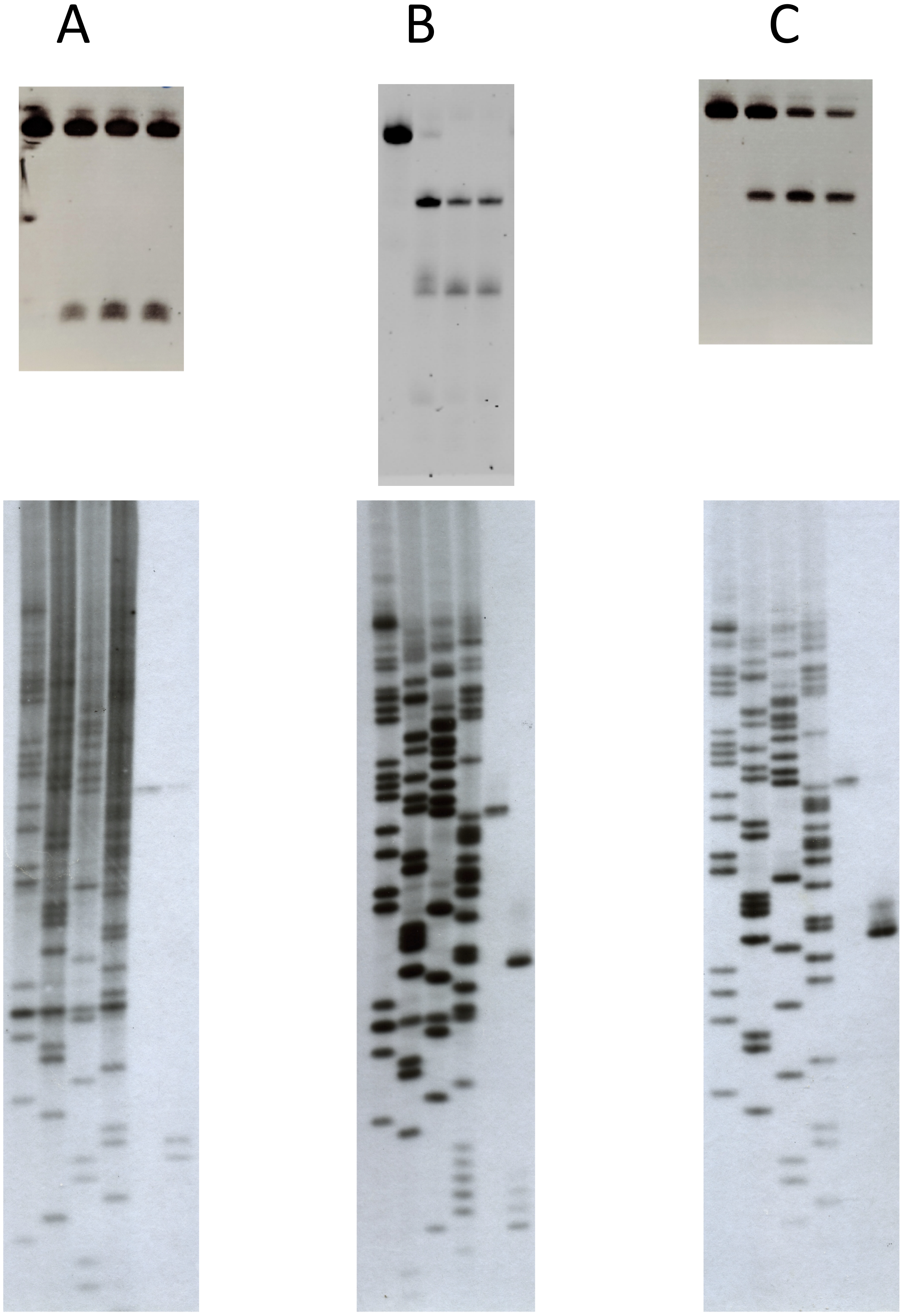

Supplementary Information Figure 8: Uncropped Images from Figure 8
